# Detecting Faults at the Edge via Sensor Data Fusion Echo State Networks

**DOI:** 10.3390/s22082858

**Published:** 2022-04-08

**Authors:** Dario Bruneo, Fabrizio De Vita

**Affiliations:** Department of Engineering, University of Messina, 98166 Messina, Italy; fdevita@unime.it

**Keywords:** ESN, recurrent neural networks, sensor data fusion, edge computing, industry 4.0, fault detection, deep learning

## Abstract

The pervasive use of sensors and actuators in the Industry 4.0 paradigm has changed the way we interact with industrial systems. In such a context, modern frameworks are not only limited to the system telemetry but also include the detection of potentially harmful conditions. However, when the number of signals generated by a system is large, it becomes challenging to properly correlate the information for an effective diagnosis. The combination of Artificial Intelligence and sensor data fusion techniques is a valid solution to address this problem, implementing models capable of extracting information from a set of heterogeneous sources. On the other hand, the constrained resources of Edge devices, where these algorithms are usually executed, pose strict limitations in terms of memory occupation and models complexity. To overcome this problem, in this paper we propose an Echo State Network architecture which exploits sensor data fusion to detect the faults on a scale replica industrial plant. Thanks to its sparse weights structure, Echo State Networks are Recurrent Neural Networks models, which exhibit a low complexity and memory footprint, which makes them suitable to be deployed on an Edge device. Through the analysis of vibration and current signals, the proposed model is able to correctly detect the majority of the faults occurring in the industrial plant. Experimental results demonstrate the feasibility of the proposed approach and present a comparison with other approaches, where we show that our methodology is the best trade-off in terms of precision, recall, F1-score and inference time.

## 1. Introduction

The emergence of the Industry 4.0 paradigm has made modern plants involve several set of sensors to enable monitoring under many different aspects. The pervasive presence of sensors and actuators in industrial systems totally changed the interactions with these systems and paved the way for the realization of more precise control and telemetry frameworks [1]. On the other hand, when the number of sensors is large, it becomes difficult to correlate and analyze the information coming from several heterogeneous data sources and produce an accurate diagnosis of the system’s “health”. In particular, dealing with signals that can be very different (e.g., in terms of sampling rate, data format, protocols, etc.) represents one of the major challenges during the selection process of the more informative signals [2].

In such a context, the use of Artificial Intelligence (AI) techniques based on sensor data fusion can be considered a viable solution to address this problem. Through machine and deep learning approaches, we realize intelligent algorithms capable of separately extracting features from a set of heterogeneous signals and then fuse this information to improve the predictive performance [3].

Cloud computing paradigm has played a key role in smart factories, acting as a central entity providing the storage and computing power necessary for the execution of complex algorithms [4]. However, when working with industrial applications aiming to predict the state of a system, time becomes a crucial component. Indeed, being able to perform a timely detection of a fault condition can avoid more severe consequences while having a strong impact in terms of time and money.

Today, Edge computing became a common solution to address this problem by shifting the computation closer to the data, thus improving the security, response times, and connection stability [5]. As a side effect, the use of this paradigm poses significant restrictions on the type of algorithms that can be performed. In this sense, an Edge device is typically equipped with a constrained hardware with limited resources, which makes it unsuitable for the execution of advanced algorithms (e.g., Deep Neural Networks) in an effective way [6].

Reservoir Computing (RC) [7] is a promising framework deriving from the Recurrent Neural Networks (RNNs) characterized by a container (called *reservoir*) that remains fixed during the training procedure and whose task is to learn complex input dynamics [8,9]. Among the models belonging to the RC, Echo State Networks (ESNs) are a family of neural networks particularly suitable for the analysis of time series, which embody the power of RNNs into models with a low number of trainable parameters [10], thus exhibiting a reduced memory footprint and complexity that can meet the strict requirements of an Edge device. In the context of fault detection and diagnosis, ESNs have demonstrated their effectiveness. Most of the approaches available in the literature involve the use of RNNs models for the analysis of time series. However, some RNNs suffer from unstable gradient problems that can affect the training process; ESNs address this issue by keeping the fixed weights contained in the reservoir and training only the output ones [11].

In this paper, we propose an ESN model which exploits sensor data fusion to extract the dynamics from a set of heterogeneous sensors and to perform a fault detection of a real-scale replica industrial plant. Through the implementation of a Cloud dashboard, we are able to collect and label the data generated by the plant and orchestrate the Edge devices attached to it to perform a real-time prediction. The experimental results demonstrate that the proposed approach benefits from the merging of separate source of information to improve its predicting capabilities while keeping the number of parameters low thanks to the use of an RC model. We also show the effectiveness of our solution when compared with other approaches.

The paper contributions are manifold. (i) We propose a fault detection model which exploits sensor data fusion running on a real industrial testbed. (ii) We adopt ESNs such that the proposed algorithm is suitable to be deployed on the constrained hardware of an Edge device. (iii) We realize a Cloud/Edge continuum architecture that allows the management of the data produced by the industrial system and the orchestration of the Edge devices executing the fault detection. (iv) We produce a comparison with other machine/deep learning methods in terms of predictive performance, model complexity and inference time.

The rest of the paper in organized as follows. Section 2 reviews the literature. Section 3 provides a description about the concepts at the base of ESNs. Section 4 presents the proposed proposed approach we implemented to assess the health condition of the industrial plant. Section 5 describes the experimental results we obtained from testing our technique. Section 6 concludes the paper and provides some insights for the future works.

## 2. Related Works

Sensor data fusion is becoming an important resource for the realization of smart systems. Today, we are living in an era where data of various types coming from a set of multiple sources are becoming more and more frequent, making the realization of data fusion approaches a priority. In a context characterized by a multitude of information, the swarm intelligence paradigm is another important player for extracting important features from a given dataset [12] or to solve high-dimensional optimization problems [13]. In this section, we review the works found literature, and we highlight the main differences with our solution.

The authors in [14] present a review about sensor data fusion methods for real-time analysis, putting in evidence the main concepts, the core steps and the research challenges.

In [15], the benefits that derive from the use of sensor data fusion as an effective technique to produce more accurate and reliable results through the combination of data coming from multiple sources are described. In this work the authors also present diverse data fusion schemes, highlighting the differences among them.

In [16], a Deep Enhanced Fusion Network (DEFN) is proposed for the fault diagnosis of wind turbine gearboxes using three-axial vibration signals. In this work, the authors adopt Sparse Autoencoders (SAEs) to perform the feature extraction process, and such features are then passed to an ESN to perform the actual fault detection. Although the obtained results are good, the technique is not meant to be run on the Edge due to the model’s complexity. In this sense, our model has been designed to run on constrained devices thanks to its low number of trainable parameters.

The authors in [17] describe a multisensor approach for chattering detection in milling processes. Starting from sound and vibration signals, they are passed to several feature extraction processes such as Wavelet Package Decomposition (WPD), WPD parameters optimization, and the extraction of time/frequency features. Then, these features are passed as input to an ensemble method made of five algorithms, namely, Random Forest (RF), Extreme Gradient Boost (XGBoost), Support Vector Machines (SVMs), K-Nearest Neighbor (KNN) and Artificial Neural Network (ANN), to perform the chatter identification. The experimental results demonstrated the effectiveness of the authors approach; however, the large number of processing steps together with the use of an ensemble method produce a bottleneck during the inference time. When working in fault detection applications, the response time is a key factor. Our approach benefits from the use of ESNs, which exhibit a fast inference time suitable for industrial applications.

In [18], the authors propose a vibro-acoustic data fusion approach based a one-dimensional Convolutional Neural Network (1D-CNN) for bearing fault detection. The model presented in this work adopts CNNs to separately extract the features from vibration and acoustic signals. Then, these features are combined together and passed to a series of fully connected layers that perform the feature fusion. The approach obtained very good results; however, compared to ESNs, CNNs require a larger number of trainable parameters, which could not fit the memory requirements. Moreover, if not properly parametrized CNNs are more prone to overfitting. A similar approach is described in [19], where the authors adopt a 2D-CNN to fuse data coming from multiple current sensors and to perform an automatic feature extraction process. Although the results obtained are good, these method suffers from the same problem highlighted in [18].

The work presented in [20] makes use of a sensor data fusion approach to predict the Remaining Useful Life (RUL) of electromagnetic pumps. Given the pressure and vibration signals collected from the pump, the prognostic algorithm adopts a multi-objective genetic algorithm based on Long Short-Term Memories (MOGA-LSTM). The authors have successfully demonstrated the effectiveness of their technique, but exactly as in the previous cases, this algorithm is not designed to be executed of an Edge device. In our application context, this aspect is of fundamental relevance in terms of quick response times, privacy and security.

In [21], a sensor data fusion algorithm based on NARX neural networks is proposed to predict the mass flow in sugarcane harvesters. In this case, the input data are passed to a single hidden neuron for the feature extraction process; if, on the one hand, this reduces the model complexity, on the other, it does not exploit the full potential of sensor data fusion that allows it to analyze each input separately to achieve a tailored feature extraction. Of course, when the number of inputs is large, this could lead to models with a lot of layers and parameters. To mitigate this effect in our solution, we adopted ESNs, which keep fixed the majority of their weights.

The authors in [22] discuss the misalignment fault classification and describe a solution based on SVMs and vibro-acoustic sensor data fusion. Such data are used to analyze the system under different load conditions and operative settings and reached a 100% accuracy in detecting the faults. If, on the one hand, the authors obtained impressive results, on the other, the use of SVMs does not allow for catching time dynamics that only an RNN is able to catch.

In [23], a work for gear fault classification in rotating machines is presented. Starting from a set of vibration sensors, the models adopts Coherent Composite Spectrum (CCS) for a first feature extraction. Then, Principal Component Analysis (PCA) and ANN are used to perform the dimensionality reduction and a further feature extraction. These are then merged and passed to a fault classification block for the actual prediction. The authors demonstrated the effectiveness of their approach and the benefits derived from the use of PCA that allowed them to reach a 100% accuracy.

## 3. Echo State Networks

**Figure 1:**
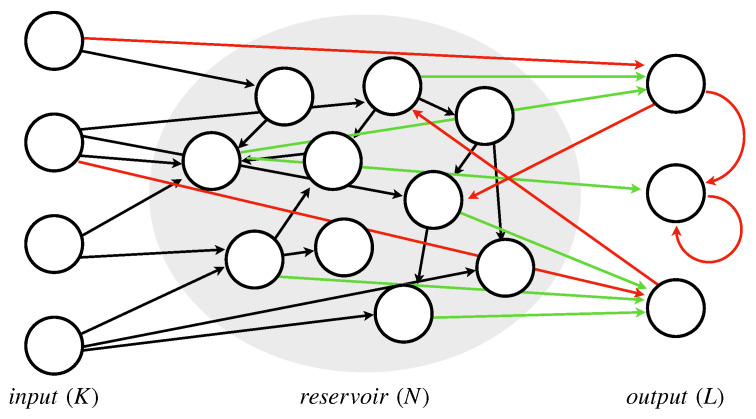
ESN architecture.

ESNs are a family of neural networks which belong to the RC models class particularly suitable for the analysis of time series data [24,25]. From an architectural point of view, an ESN is equivalent to an RNN, except for the presence of a sparse and randomly connected recurrent structure called a reservoir performing the feature extraction process. A peculiar aspect of this layer is the fact that the reservoir weights are fixed and do not change during the training process. Figure 1 depicts an ESN architecture, where the green and the black arrows are the trainable and the not-trainable weights, respectively, while the red lines indicate possible, but not required, connections.

Given an ESN with *K* input units, *N* reservoir units and *L* output units, we can define the equations which govern the ESN as follows:

(1) $$x\left(t+1\right)=f(W_{in}\cdot u\left(t+1\right)+W_{r}\cdot x\left(t\right)+W_{b}\cdot y\left(t\right)),$$

(2) $$y\left(t+1\right)=g(W_{out}\cdot \left[u\left(t+1\right),x\left(t+1\right),y\left(t\right)\right]),$$

where x(t+1) is the new computed reservoir state and is a function of the new input u(t+1), the state x(t) and the output y(t). As far as $W_{in}$, $W_{r}$ and $W_{b}$, they are matrices of N×K, N×N and N×L elements, which store the weights between the input and reservoir layers, the reservoir weights and the backwards connections between the output and reservoir layers, respectively. Finally, f(·) is the activation function wrapping the equation (typically the sigmoid or the tanh).

With respect to the output equation, it is obtained as the concatenation of the new state x(t+1), the input u(t+1) and the previous output y(t); in this sense, the output is “affected” by the past history of the model. The $W_{out}$ is an L×(K+N+L) matrix containing the only trainable weights of the network (i.e., those connecting the reservoir layer to the output). Also in this case, an activation function g(·) (typically a sigmoid or the identity) wraps the equation to generate the actual output of the network.

Compared to other RNN models such as Gated Recurrent Units (GRUs) and LSTMs, ESNs can exploit the sparse recurrent structure of reservoir layer to achieve comparable results using much less parameters. In this sense, a model with a reduced number of parameters is less prone to overfitting, a well-known problem when working with complex Deep Neural Networks (DNNs), where a huge number of parameters produces large models. Evidently, this involves a reduction in the model memory footprint, a key aspect that should not be underestimated especially in this period where the majority of applications is migrating towards the constrained hardware of Edge devices. Another advantage of ESNs is represented by their faster training/inference time thanks to the fact that the majority of the weights are kept fixed. Considering our industrial scenario where the system response time is a crucial requirement, together with a small model footprint such that it can fit the hardware of an Edge device, the above-mentioned features motivated the choice of ESNs as a preferable approach over other solutions.

However, if, on the one hand, the sparse and fixed reservoir weights allow reducing the ESN model complexity and speed-up the training process due to the reduced number of parameters, on the other, the impossibility of optimizing these weights can lead to unstable results due to the random initialization [26]. Such an effect can be mitigated by using large reservoir structures to incentivize the creation of subnetworks that can catch a large number of input dynamics [8,24], but this still remains one of the main limitations of these networks.

When working with ESNs, it is also very important to satisfy the *separation* and *echo state* properties to make them properly work. With respect to the first one, it ensures the generation of separate states given different inputs. Such a condition is fundamental to avoid the extraction of the same patterns for several inputs that would inevitably cause the collapse of the ESN. In this sense, the adoption of large and sparse reservoirs is necessary to meet this property by “encouraging” the formation of mixed connections with the input layer that stimulate the production of different states and features. The echo state property (from here, the name of these networks) states that the effects of the inputs and the previous states in Equation (1) should gradually vanish over time. Indeed, this is a very important feature for an ESN since it allows balancing the present and past history of the model, so that the output is equally affected. Unlike the previous case, the satisfaction of this property comes from empirical tests, where it has been demonstrated that a $W_{r}$ weight matrix with a spectral radius (i.e., the largest eigenvalue in absolute value) lower than one is a necessary (but not sufficient) requisite. The use of weight scaling techniques produce suitable matrices which meet this condition; however, it is worth to mention that for specific types of inputs, this could be neither necessary nor sufficient. For this reason, the echo state property is still the object of study.

## 4. Proposed Data Fusion Approach

In this subsection, we introduce the scale replica plant used as an industrial testbed, and we present the proposed approach which merges sensor data fusion and ESNs to assess its working conditions.

### 4.1. Industrial Testbed

The industrial testbed we used is a scale replica of an assembly plant for the transportation of car pieces adopted in automobile factories. It is powered with two gear motors, a set of six belts, and a mobile cart that can move back and forth. We instrumented the plant with vibration and current sensors to enable a mechanical and electrical monitoring, and we equipped it with a set of Edge devices for the real-time execution of our fault-detection algorithm. In particular, we used a VTV-122 sensor by IFM electronics to measure anomalous vibrations due to the brake system or cart proximity switch malfunctioning. The sensor operates with a power supply between 9 and 32 V and outputs a 4–20 mA signal. For the electrical part, we used an inverter of the S100 series by LS Industrial Systems that we directly attached to one of the gear motors. The inverter uses a power supply between 200 and 240 V and generates an analog current output in the range 0 to 20 mA. On the Cloud side, we realized a platform, accessible via a web dashboard, through which we can collect and label the data produced by the sensors and orchestrate the Edge side deploying new deep learning models, thus creating a Cloud/Edge continuum architecture.

In such a context, the possibility to manually inject faults in our scale replica testbed (e.g., the introduction of external vibrations along the plant structure, the failure of the brake system, the increment in gears friction, the change of belts tension, etc.) has been fundamental to enable the data labeling of anomalous patterns, and gave us the opportunity to study the testbed also when subject to a faulty condition.

**Figure 2:**
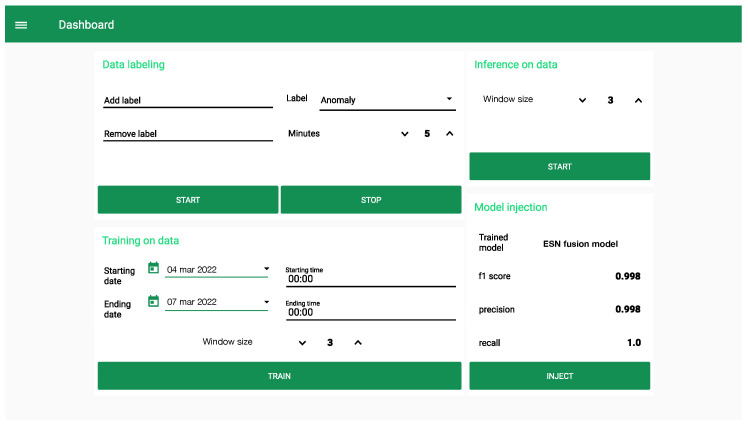
Dashboard running on the Cloud to orchestrate the Edge devices.

Figure 2 depicts the Cloud dashboard we realized to orchestrate the Edge devices connected to the industrial plant. It is divided into four sections. The first one allows the labeling of the data collected through industrial plant and their storage under different operating conditions of the system. The second one defines the starting and ending dates and time that the system will use for making a query to the internal Cloud database and to retrieve the training data. At this step, the user can also define the windows size to split the input signals into smaller time sequences to be analyzed. The third section is used to start the inference at the Edge in order to assess the working conditions of the industrial plant. Here the window size performs the same task as in the training process. Finally, the fourth section returns a report of the trained model by showing its performance and informing the user which model is going to be injected at the Edge.

### 4.2. Fault Detection

Catalyzed by the advent of the Industry 4.0, modern systems faced a complete transformation that revolutionized the way to interact with them. Today, we can observe industrial plants that are equipped with a large number of sensors and actuators, becoming a “smart entity” with a self-awareness that can help the human operator during the execution of several tasks. In such a context, one of the most important aspects is the diagnosis of industrial systems conditions, which became a central research topic. Preventive maintenance is no longer an option that can considered: The huge amount of components in industrial plants makes the use of this approach unsustainable, and for this reason, modern solutions are moving towards the realization of predictive frameworks capable of detecting abnormal behaviors before the occurrence of the fault.

However, when working with complex systems, the generation of an accurate diagnosis can be very challenging due to strong nonlinearity, data heterogeneity and the large number of process variables to take into account. Deep learning can address this problem through the definition of advanced predictive models that can manage a large number of inputs and learn hidden relationships among them. Fault detection represents a key element to recognize harmful patterns that usually anticipate a failure, thus reducing the maintenance costs of a system [27].

Along with fault detection, it is worth mentioning the crucial role played by Edge computing; the wide spread of this technology (which rapidly became the core element of smart factory frameworks) is a perfect example that proves the effectiveness of this paradigm. In a context where a large part of the tasks operated by an industrial system is executed in real time emerges the necessity to perform specific analyses very close to where the data are generated. Also in terms of security, Edge computing can be very useful to better preserve data privacy by performing the inference process on data stored locally. With particular reference to the industrial scenario, where the data can be sensitive, the capabilities of this technique allow the realization of more secure applications. However, moving the computation towards these devices poses significant limitations on the realization of algorithms that should meet their strict hardware resources to run effectively.

### 4.3. Sensor Data Fusion Model

**Figure 3:**
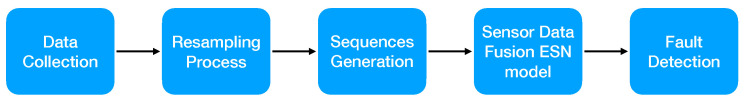
Block diagram showing the steps performed in our framework.

The fault detection problem has been faced as a supervised binary classification problem where we considered two possible working states for the plant, namely, a normal condition and an anomalous one. Figure 3 shows a block diagram with the main steps performed in our framework. The data collection methodology we adopted is the following. In order to have a monitoring from both the electrical and mechanical point of view, we instrumented the plant with a vibration sensor directly attached on its structure and a inverter that we connected to one of the engines to measure the absorbed current. With respect to the sampling rates, we set the vibration signal to 30 Hz, which, from the empirical tests, was demonstrated to be a good trade-off in terms of computational complexity and signal reconstruction. Regarding the inverter, we set its internal sampling rate to 120 Hz in order to meet the Nyquist frequency; however, we noticed that the absorbed current remained stable for the majority of the time, exhibiting a change only when a fault was occurring. Considering that these signals are also analyzed in real time by an Edge device with limited computing capabilities, working with such a high rate would be unfeasible. For these reasons, we created a script to interrogate the inverter with a 5 Hz rate. The data collection phase lasted about 46 h; we started collecting the data under a normal operating setting for about 29 h, in which the plant was properly working and the cart was able to move back and forth. After this step, in the remaining 17 h, we started the injection of the anomalies (for about 8.5 h each and one at a time) inside the system, first increasing the friction of the gears and then changing the belts’ tension. At the end of this process, we collected ∼5 M of vibration samples (i.e., ∼3 M normal, and ∼2 M anomalous) and ∼800 K current samples (i.e., ∼5 K normal, and ∼3 K anomalous).

**Figure 4:**
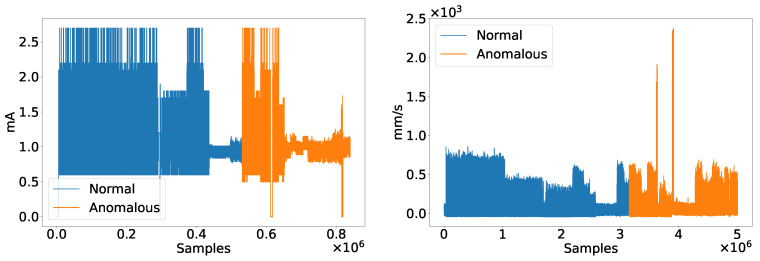
Current (**left**) and vibration (**right**) raw signals collected from the industrial plant in correspondence of the normal and anomalous conditions.

Figure 4 depicts the current and vibration signals collected from the industrial plant in correspondence of the two above-mentioned conditions. The first problem we faced working with these signals has been to make them have the same sampling rate. Specifically, we adopted the SciPy Python library, which exposes some utility APIs for signals resampling; in this sense, since the vibration exhibited a much higher number of samples than the current, we performed an upsampling process on the last one. When working with time series, having a homogeneous sampling rate is fundamental to avoid a time uncorrelation between signals that would inevitably affect the overall prediction performance.

Then, we performed a second data preprocessing to split the raw signals into multiple time sequences by applying an overlapping sliding window. As an effect of this step, we were able to reduce the inputs complexity by focusing on smaller parts, in addition, the analysis of several sequences instead of the whole signal allows a better feature extraction. The choice of the window size strongly depends on the signals characteristics (e.g., sampling rate, variability, frequency, etc.); in our empirical experiments, we tested several windows values using as evaluation indices the resulting model prediction performance and model complexity. At the end of this step, we selected a window size of 50 samples for the input sequences that resulted to be the best trade-off. At the end of these preprocessing steps, data were ready to be passed to the sensor data fusion model.

**Figure 5:**
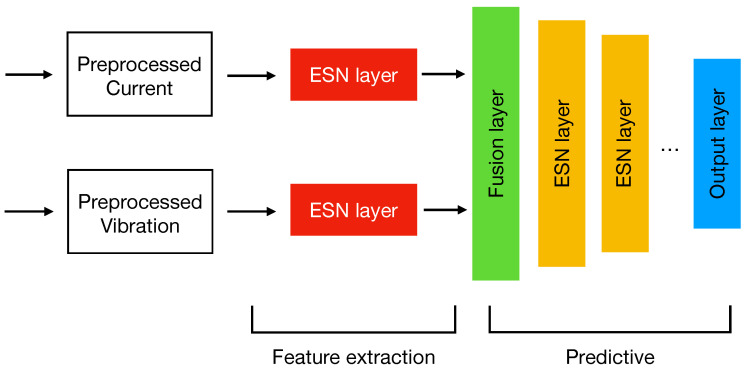
Proposed Sensor data fusion ESN model.

Figure 5 shows the proposed sensor data fusion ESN model. It is structured into two parts: a feature extraction and a predictive one. Unlike a “traditional” model, where input data are typically treated as a single monolithic block, in this case, we can notice that each signal is separately managed and passed to an ESN. Such a separation has two effects: (i) It improves the model input scalability, since the addition of new signals requires a change only in the feature extraction part and not of the entire topology; (ii) it allows a tailored feature extraction according for each input. In such a context, where inputs are time series signals, the use of RNN structures represent a valid solution to find hidden time dynamics that a normal machine learning model would not be able to catch. However, when working with recurrent models such as LSTMs or GRUs, they are prone to generating a lot of parameters and require a careful parametrization. In this sense, the choice of ESNs resulted in being a good option since they work very well as time features extractors [28] and use a low number of trainable parameters which make them lightweight, fast to train and suitable to fit the hardware of an Edge device. At this step, each ESN analyzes only one signal separately from the other one; by means of their reservoir, they are able to extract from the input sequences a hidden vector containing the temporal features. The main idea behind this model is that the reservoir represents a latent space. In fact, thanks to its large dimensionality, this non-linear part of the system can extract relevant features (at a higher abstraction layer) deriving from the input series.

The predictive part of the model starts with the Fusion layer which acts as a connecting bridge between the input branches. At this level, the features extracted in the previous layers are concatenated together performing the actual sensor data fusion. Finally, the ESN layers connected in cascade have the task to perform a further feature extraction process necessary for the prediction whose results are stored in the output layer. According to the prediction task, the output layer can have various shapes and adopt different activation functions. For example, in the case of a multiclass classification problem, the output layer will have as many neurons as the number of classes to be predicted and will adopt a *softmax* activation function to produce the probabilities for a given input to belong to each of them. On the other hand, if the task to be addressed is a binary classification, then in this case is sufficient to use as output a single neuron with a sigmoid activation to evaluate the class to which the input belongs to.

Let us define the mathematical model of our sensor data fusion ESN. If we consider a model with *m* separate inputs, then we can write the state and output equations of the generic *i*th ESN as follows:

(3) $$\left\{\begin{array}{c} x{\left(t+1\right)}^{i}=f\left(W_{in}^{i}\cdot u{\left(t+1\right)}^{i}+W_{r}^{i}\cdot x{\left(t\right)}^{i}\right)\\ h{\left(t+1\right)}^{i}=f\left(W_{h}^{i}\cdot x{\left(t+1\right)}^{i}\right), \end{array}\right.$$

where the equations reported in Equation (3) are a variation of the ones shown in Section 3 and obtained by removing the not required connections (i.e., the red arrows of Figure 1). In such a context, the output of each ESN is a hidden vector $h{\left(t+1\right)}^{i}$ storing the temporal features extracted from the reservoir layer. Unlike Equation (2), in this case, the $W_{h}$ matrix does not contain trainable weights as the output of the ESN is in turn a hidden vector:

(4) $$\left\{\begin{array}{c} F\left(t\right)=[h{\left(t+1\right)}^{1},h{\left(t+1\right)}^{2},\ldots ,h{\left(t+1\right)}^{m}]\\ X\left(t+1\right)=f(W_{F}\cdot F\left(t+1\right)+W_{R}\cdot X\left(t\right))\\ y\left(t+1\right)=\phi (W_{out}\cdot X\left(t+1\right)). \end{array}\right.$$

In Equation (4), we report the equations modeling the predictive part, where F(t) is the output of the fusion layer obtained as the concatenation of the features extracted in previous part. Since we adopted ESNs also in this part of the model, the equations are equivalent to the ones used in Equation (3), with the main difference being that, in this case, the input is no longer represented by the input signals (e.g., current and vibration) but by the features extracted from them. This is evident from the second equation, where the new computed state X(t+1) depends on F(t+1) (i.e., the features extracted and fused together at the t+1 timestep) and on the state X(t). $W_{F}$ and $W_{R}$ matrices play the same role of $W_{in}$ and $W_{r}$ of Equation (1), and for this reason, their weights are kept fixed. Finally, y(t+1) represents the actual output of the sensor fusion model, storing the prediction value; it is equivalent to the output of an ESN since it only depends on the new computed state X(t+1) and the $W_{out}$ matrix containing the only trainable weights of the entire architecture. As usual, the entire expression is wrapped by an activation function ϕ, which can be either the softmax or sigmoid based on the classification problem being addressed.

## 5. Experimental Results

In this section, we present the results obtained from testing the proposed sensor data fusion ESN model. The dataset extracted from the industrial plant is provided with binary labels such that normal and anomalous samples are labeled as 0 and 1, respectively. The splitting of the dataset has been performed using the Scikit-learn framework [29] by means of the train_test_split function, which takes as input the data and the test size percentage and returns the dataset split into train and test sets. In our experiments, we adopted a test size of 20%, leaving the rest (i.e., the 80%) for the training process. For the hyperparameters’ tuning and validation, we extracted 15% from the training data so that the test set contains samples used exclusively for the model’s evaluation. Moreover, it is worth mentioning that the following results have been averaged over a set of experiments in order to obtain a more precise evaluation.

For a better understanding, in Table 1 we report the proposed model configuration adopted in our experimentation. Such parameters derive from a grid search approach implemented using Keras Tuner (https://www.tensorflow.org/tutorials/keras/keras_tuner, accessed on 14 March 2022), during which we evaluated the network predictive performance in terms of accuracy when varying the number of ESN neurons in each layer. With respect to the feature extraction part, we used 2 ESNs (one for each input) with 20 neurons and the tanh as the activation function (representing the standard for these type of networks). Regarding the prediction part, it is composed by 2 ESNs connected in cascade with 16 and 4 neurons, respectively, with the tanh as activation. In both cases, we adopted a spectral radius of 0.9 and a connectivity of the 10% in order to meet the separation and echo state properties (see Section 3). For the trainable weights’ optimization, we used the Adam optimizer with a learning rate of 0.001, and we set the limit of the training epochs to 2000. Moreover, to avoid model overfitting, we used an early stopping approach to halt the training process if the model is not able to reduce the loss for a consecutive number of epochs defined by the patience term that we set to 10. Such a technique avoids a network “overtraining” that would inevitably cause the memorization (instead of the learning) of the input–output relationship and a consequent reduction in the model’s generalization capabilities. Finally, the output layer is defined by a single neuron associated with a sigmoid activation function to perform the binary classification.

**Figure 6:**
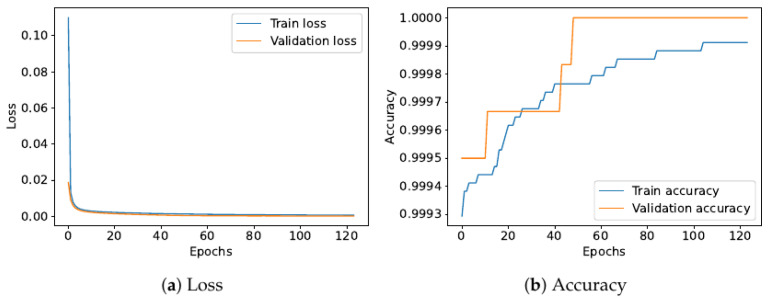
Validation curves extracted from the proposed model after the training process. In (**a**), we can observe that both the train and validation losses follow a decreasing trend as the number of training epochs increases. Both the curves converge at a very low value of the loss around zero, thus demonstrating that the model correctly learned the relationship between the input and the output with a very good level of generalization. Such a condition is also proven by (**b**), where, in this case, the training and validation accuracy increase with the training epochs, reaching very high values around one.

Figure 6 depicts the validation curves derived from the training procedure of the proposed sensor data fusion ESN model.

The proposed approach performed very well on the test set producing results comparable to the ones obtained during the training. With a precision of 1.0, a recall of 0.993 and a F1-score of 0.996, our solution is able to correctly predict the conditions of the testbed with a very low number of false positives and false negatives, a very important aspect for an algorithm running in an industrial plant. The use of ESNs for both the feature extraction and prediction parts result in an effective choice that allowed us to maintain a low amount of trainable parameters (i.e., 201) while achieving a good level of predictive performance. This, of course, has an impact not only in terms of memory footprint but also in terms of training performance that could be affordable even for an Edge device. In this sense, being able to perform an on-device training directly at the Edge could pave the way to industrial application scenarios where the plant would learn in real time the occurrence of new fault patterns.

To demonstrate the effectiveness of the proposed approach, we performed a series of comparisons with other models and techniques. Specifically, we considered six different approaches, namely: a model involving both ESN and fully connected layers (ESN + FC) which does not adopt sensor data fusion, a One Class Support Vector Machine (OCSVM) approach, an Isolation Forest (IF) model, a fully connected DNN (FC-DNN), a 1D-CNN approach like the one presented in [18] and an LSTM network. All the models have been implemented using TensorFlow and Scikit-learn frameworks [29,30].

For a better vision, we report in Table 2 the results for each of the above-mentioned models in terms of precision, recall, F1-score and the model complexity according to the number of trainable parameters.

With respect to the ESN+FC model, we implemented it using an architecture similar to the one shown in Figure 5. In particular, we used a single ESN of 40 neurons and the tanh as activation. Since this model does not adopt sensor data fusion, both the current and vibration signals are treated as a single block and passed to the ESN to perform the feature extraction process. For the prediction part, we used 3 fully connected layers of 16 and 4 neurons with Rectified Linear Unit (ReLU) as activation and a single neuron (i.e., the output) with sigmoid as activation. The model achieved very good results in terms of precision, recall and F1-score (i.e., 1.0, 0.976 and 0.987, respectively), comparable to the ones reached by our model, but using 925 trainable parameters.

For the OCSVM, Scikit-learn exposes several parameters that can be set. In our experiments, we set a penalization term (i.e., an L2 penalty) *C* of 0.8, and we left the gamma coefficient to “auto”, a special value of the library that automatically sets this coefficient to 1/(#features). The most important parameter is the kernel, which specifies the type of transformation to be used by the algorithm when fitting the training data. Scikit-learn proposes different alternatives such as linear, polynomial, radial-basis (RBF) and sigmoid. From our tests, the RBF returned the best results with a precision, recall and F1-score of 0.753, which, however, are sensibly lower than the other approaches. In terms of trainable weights, neither the algorithm nor the library provide this information, and for this reason, we did not report it in Table 2.

The IF required a lower number of parameters to be set. To select the best number of estimators to be used in the ensemble, we performed multiple experiments when varying this parameter in the range 5–100 with a step of 5. What we noticed is that the performance remained (on average) very similar with few oscillations starting from 50 estimators, and for this reason, we adopted this value. The model reached a good level of precision equal to 0.843 but resulted in the worst recall and F1-score among all the approaches with 0.294 and 0.435, respectively. Like the OCSVM, the algorithms do not make use trainable weights to execute.

With regards to the FC-DNN, in this case, our goal was to test the performance of a model not having recurrent connections and therefore unable to detect time relationships. For the realization of this topology, we used 5 hidden layers of 64, 32, 16, 4 and 1 neurons connected in cascade using the ReLU as the activation function, except for the output neuron, which adopts as usual the sigmoid. The obtained results are comparable with the ones reached by the first ESN model with a precision of 0.992, a recall value of 0.973 and a F1-score of 0.982. On the other hand, it resulted in being the model with the second largest number of trainable parameters (i.e., 2873) due to the only presence of fully connected layers which contain a lot of parameters to be trained, thus making it unsuitable for the hardware resources of an Edge device.

We also realized a model exploiting sensor data fusion LSTM, which represent the state-of-the-art in terms of RNNs. To make a fair comparison, in the feature extraction part, we used 2 LSTMs (i.e., 1 for the current signal and 1 for the vibration) with 20 neurons and tanh activation function. With respect to the predictive part, we used 2 LSTMs connected in cascade with 16 and 4 neurons with tanh activation and terminated by a layer with 1 neuron using the sigmoid. Although the LSTM reached the same results achieved by the proposed method, it resulted in being the largest model with 7705 trainable parameters. Such a result proves the power of this type of RNN, which, however, requires the optimization of a huge number of variables.

In the 1D-CNN model, each input is passed to an one-dimensional convolutional layer with 20 filters, a kernel size of 3, a stride of 1 and ReLU as the activation function. The features extracted at this stage are then passed to 2 fully connected layers connected in cascade with 16 and 4 neurons and with ReLU as activation function. Also in this case, the output layer consists of one neuron using the sigmoid to perform the binary classification. The overall performance reached by the model was good, with a precision of 1.0, a recall of 0.994 and a F1-score of 0.996; on the other hand, it resulted in being the third largest model after the FC-DNN and LSTM models with 1077 trainable parameters.

As already mentioned, time is a crucial component, especially in fault detection applications, where a prompt response can save a system from a total breakdown. To this aim, we conducted a comparison of the models average inference time, focusing our attention only on the neural network models which returned the best performance when compared with IF and OCSVM approaches.

**Figure 7:**
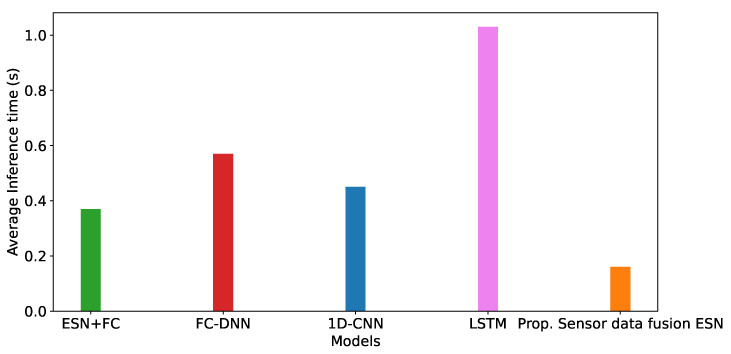
Models’ average inference time.

Figure 7 shows the average inference time of the five neural network models we considered in our experimentation. With respect to the ESN + FC model, it reached an inference time of 0.37 s; in such a context, we can observe that the use of the ESNs in the feature extraction part is beneficial, making this approach one of the fastest. The FC-DNN is one of the slowest models due to its large number of trainable parameters, with an average inference time of 0.57 s. Regarding the 1D-CNN, we observed a slight improvement with respect to the DNN with an inference time of 0.45 s. From an architectural point of view, this model is very similar to the ESN+FC one: They share, in fact, the same number of fully connected layers, the same neurons and the same activation functions. The only difference is represented by the feature extraction process, which is performed through an ESN and a convolutional layer. Such a result, further demonstrates the effectiveness of ESNs in reducing the inference time thanks to their sparse structure and low number of parameters. As we would expect, the LSTM model resulted in being the slowest model, with an average inference time of 1.03 s. Finally, the proposed sensor data fusion ESN, which in the previous analysis exhibited the lowest amount of parameters, in this case, resulted in being the fastest among the other models with an average inference time of 0.16 s.

The results derived from these comparisons demonstrate the effectiveness of the proposed approach, which reached a good level of performance comparable with the ones achieved by other neural network models while using a very low number of trainable parameters and exhibiting the fastest inference time. The combination of these results makes our model sufficiently small to be deployed on an Edge device with a response time suitable for the detection of faults in an industrial application context. However, we should point out that even if nowadays most of the industrial plants are provided with historical databases, it can happen that the data labeling process it not available or possible. In this sense, the supervised nature of the proposed approach poses a limitation to those contexts where it is possible to gather labeled data.

## 6. Conclusions

In this paper, we proposed a sensor data fusion model that exploits ESNs to perform the fault detection at the Edge of a real-scale replica industrial plant. Although the supervised nature of our method limits its applicability, the experiments demonstrated the feasibility of the proposed approach. Thanks to the use of ESNs we were able to obtain a model which achieved a very good level of performance with a precision of 1.0, a recall of 0.993 and a F1-score of 0.996, while keeping a limited number of trainable parameters. Such a result makes our approach suitable to be deployed on the constrained hardware of an Edge device; moreover, the fast inference time exhibited by our solution allows us to perform a real-time detection of faults in industrial systems.

Future works will be devoted to the improvement of the proposed technique through the realization of an unsupervised scheme capable of extracting useful features from the inputs and perform a fault detection on unlabeled data, to the use of a larger number of signals for a better plant monitoring and to the implementation of an on-device training procedure in order to enable a continuous learning application where the industrial plant can autonomously learn the emergence of new fault patters.

## Figures and Tables

**Table 1:** Proposed model configuration.

Training Parameters	
ESN neurons feature extraction part	20
ESN neurons predictive part	16, 4
ESN spectral radius	0.9
ESN connectivity	0.1
Output neurons	1
Activation functions	tanh, sigmoid
Learning rate	0.001
Training epochs limit	2000
Optimizer	Adam
Patience term	10

**Table 2:** Models’ comparison.

Model	Precision	Recall	F1-Score	#TrainableParams.
ESN+FC	1.0	0.976	0.987	925
OCSVM	0.753	0.753	0.753	-
IF	0.843	0.294	0.435	-
FC-DNN	0.992	0.973	0.982	2,873
LSTM	1.0	0.993	0.996	7,705
1D-CNN [18]	1.0	0.994	0.996	1,077
Proposed Sensor data fusion ESN	1.0	0.993	0.996	201

## Data Availability

Not applicable.
